# Supplementary material for: The function of bacterial HtrA is evolutionally conserved in mammalian HtrA2/Omi
Source: Sci Rep. 2020 Mar 24;10:5284. doi: 10.1038/s41598-020-62309-z (PMC7093540; doi:10.1038/s41598-020-62309-z)
Supplement: Supplementary file 1 — Supplementary Information. [file 41598_2020_62309_MOESM1_ESM.docx]

**Supplementary information**

**The function of bacterial HtrA is evolutionally conserved in mammalian HtrA2/Omi**

*Hea-Jong Chung, Mohammad Abu Hena Mostofa Jamal, Seong-Tshool Hong^*^*

**Contents:**

Supplementary Table 1

Supplementary Fig. 1, 2, 3, 4, 5, 6

**Supplementary Table 1. Resources used in experiments**

| Resources | | Source | | | Identifier | Purpose | |
| --- | --- | --- | --- | --- | --- | --- | --- |
| Antibodies | | | | | |  | |
| Mouse anti-α-Syn(4D6) antibody | | Abcam, Cambridge, UK | | cat# Ab1903 | | Western blot, IHC | |
| Mouse anti-HtrA2/Omi antibody (AA 134-458) | | Antibodies-online GmbH, Aachen, Germany | | cat# ABIN121159 | | Western blot | |
| Goat anti-mouse IgG (H+L) antibody | | Promega Corporation,Wisconsin, USA | | cat# W4021 | | Western blot | |
| Rabbit anti-α-Syn | | Abcam, Cambridge, UK | | cat# Ab51252 | | IHC, Confocal Microscopic study | |
| Rabbit anti- HtrA2/Omi | | Abcam, Cambridge, UK | | cat# Ab64111 | | IHC | |
| Mouse anti human α-Syn antibody (ASy05), oligomer specific | | Agricera antibodies, Vannas, Sweden | | cat# AS132718 | | IHC, Confocal Microscopic study | |
| AlexaFluor 488-conjugated goat anti-mouse IgG (H+L) | | Invitrogen, California, USA | | cat# A11029 | | IHC, Confocal Microscopic study | |
| Alexa Fluor 568-conjugated goat anti-rabbit IgG (H+L) | | Invitrogen, California, USA | | cat# A11011 | | IHC, Confocal Microscopic study | |
| Mouse anti-PDI | | Abcam, Cambridge, UK | | cat# Ab5484 | | Co-localization study | |
| Mito Tracker Red CMXRos | | Invitrogen, California, USA | | cat# M7512 | | Co-localization study | |
| Goat anti-rabbit IgG (H+L) conjugated to FITC | | Abcam, Cambridge, UK | | cat#Ab6717 | | IHC, Confocal Microscopic study | |
| Goat anti-mouse IgG (H+L) conjugated to Texas Red | | Abcam, Cambridge, UK | | cat# Ab6787 | | IHC, Confocal Microscopic study | |
| Reagents | | | | | | | |
| Horse serum | | Sigma-Aldrich, Missouri, USA | | cat# H1270 | | | IHC |
| Goat serum | | Sigma-Aldrich, Missouri, USA | | cat# G9023 | | | IHC |
| Protease inhibitor cocktail Complete mini | | Roche Applied Science, Mannheim, Germany | | cat# 11836153 001 | | | Brain homogenate preparation |
| Recombinant Human α-Syn | | r Peptide, Georgia, USA | | cat# S-1001 | | | *In vitro* oligomerization |
| UCF-101 | | Merck Millipore, Massachusetts, USA | | cat# 496150 | | | *In vitro* enzymatic assay |
| VECTASTAIN Elite ABC Kit | | Vector Laboratories Inc., California, USA | | cat# PK-6100 | | | IHC |
| Avidin/Biotin Blocking Kit | | Vector Laboratories Inc., California, USA | | cat# SP-2001 | | | IHC |
| DAB Peroxidase (HRP) Substrate | | Vector Laboratories Inc., California, USA | | cat# SK-4105 | | | IHC |
| Sephadex G-25 resign | | Sigma-Aldrich, Missouri, USA | | cat# G25150 | | | Size exclusion chromatography |
| Glass Wool | | Sigma-Aldrich, Missouri, USA | | cat# 20411 | | | Size exclusion chromatography |
| Experimental Model | |  | |  | | |  |
| Mouse:  C57BL/6J-mnd2 mice | | Jackson Laboratory, Maine, USA | | N/A | | | *In vivo* experiments |
| Fly:  α-Syn transgenic *Drosophila*  (α-Syn/ α-Syn) | | Bloomington *Drosophila* stock center, Indiana, USA | | FBst0008146 | | | *In vivo* experiments |
| Human HtrA2/Omi transgenic *Drosophila* (hOmi^+/Cyo^) | | In this study | | N/A | | | *In vivo* experiments |
| α-Syn and hOmi co-overexpressed *Drosophila* (hOmi/hOmi; α-Syn/α-Syn) | | In this study | | N/A | | | *In vivo* experiments |
| Driver line elav-GAL4 | | Bloomington *Drosophila* stock center, Indiana, USA | | FBst0000458 | | | pan neuronal expression of transgenes |
| Driver line GMR-GAL4 | | Bloomington *Drosophila* stock center, Indiana, USA | | FBst0009146 | | | Expression of transgenes in eye |
| Primers | |  | |  | | |  |
| Gene name to identify | | Forward Primer | | Reverse primer | | |  |
| α-Syn | 5’-TGT AGG CTC CAA AAC CAA GG-3’ | | 5’-GCT CCC TCC ACT GTC TTC TG-3’ | | | | |
| hOmi | 5’- GTC GCC GGA TCC ATG CGC TAC ATT-3’ | | 5’-GAG CTC TCG AGT CAT TCT GTG ACC-3’ | | | | |

**Supplementary Fig. 1** Oligomeric α-Syn was isolated using a size exclusion column. (**a**) The SDS-PAGE gel image showing oligomerized α-Syn fractionations in the size exclusion column. Human recombinant monomeric α-Syn was oligomerized. Monomer, dimer, trimer, tetramer as well as higher-order oligomers of α-Syn were distinguished by molecular weight. (**b**) The isolated oligomeric α-Syn using the size exclusion column.

**Supplementary Fig. 2** HtrA2/Omi degraded specifically oligomeric α-Syn in a dose-dependent manner. The degradation of α-Syn was measured by the fluorescence intensity after staining of α-Syn with the oligomer-specific fluorescent dye ThT. Values represent the mean ± SEM from three independent experiments. *^*^p*<0.05 and *^**^p*<0.01.

**Supplementary Fig. 3** The neurotoxicity of oligomeric α-Syn was abolished after co-treatment with HtrA2/Omi in a dose-dependent manner. Recombinant hOmi treatment was applied at 0 nM, 10 nM, 50 nM and 100 nM, and cell viability was assessed using the CCK-8 assay. Values represent the mean ± SEM from three independent experiments. NS, not significant and *^***^p*<0.001.

**Supplementary Fig. 4** A transgenic *Drosophila* line expressing human HtrA2/Omi was successfully developed. (**a**) The schematic representation of the development procedure for the transgenic *Drosophila* lines expressing human HtrA2/Omi (hOmi). The full-length hOmi gene was cloned into the GAL4-responsive pUAST expression vector. Transgenic *Drosophila* lines of hOmi were generated by microinjection of a plasmid bearing the P element transposase under the control of the heat shock 70 (hs-π) promoter to drive the expression of transposase into w^1118^ embryos. (**b**) The detection results of the hOmi gene in 4 transgenic *Drosophila* lines by PCR. (**c**) The detection results of the hOmi protein in 4 transgenic *Drosophila* head homogenates by western blotting, in which head homogenates were obtained from the progeny of hOmi crossed with elav-GAL4 flies. (**d**) Relative expression levels of hOmi in the transgenic lines. Tg4 was selected for next experiment on the basis of hOmi expression levels.

**Supplementary Fig. 5** A Transgenic *Drosophila* line homozygotely carrying both HtrA2/Omi and α-Syn genes was developed. (**a**) Schematic representation of developing a *Drosophila* line homozygotely carrying both hOmi and α-Syn genes by crossing hOmi/+ transgenic *Drosophila* with α-Syn transgenic *Drosophila.* (**b**) Example of 2 transgenic *Drosophila* lines homozygotely carrying hOmi. The *Drosophila* lines homozygotely carrying hOmi were identified by genotyping of the fly progenies. If all of the progenies from either parent crossed with control carried hOmi, the parental flies were identified as homozygotes and used in this work. (**c**) Example of 2 transgenic *Drosophila* lines homozygotely carring α-Syn. The *Drosophila* lines homozygotely carring α-Syn were assessed by genotyping of the fly progenies. If all the progenies from either parent crossed with the control had α-Syn, the parental flies were identified as homozygotes and used in this work.

**Supplementary Fig. 6** The Kaplan–Meier Survival assay on the hOmi, α-Syn, or hOmi/α-Syn flies demonstrated a neuroprotective role of HtrA2/Omi against α-Syn-induced cytotoxicity in both male (**a**) and female (**b**) flies. Values are the mean ± SEM from three independent experiments. NS, not significant, *^*^p*<0.05, *^**^p*<0.01, *^***^p*<0.001.

**Figure S1.**

**
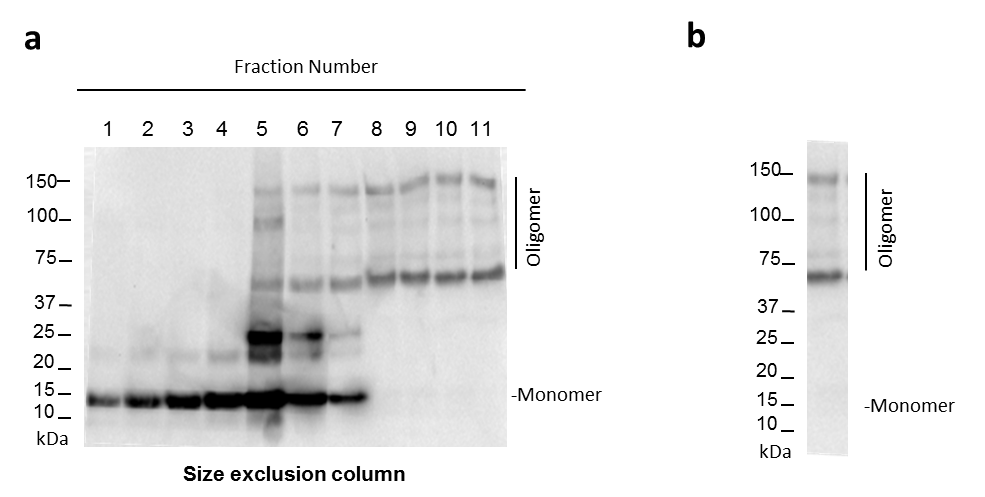
**

**Figure S2.**

**
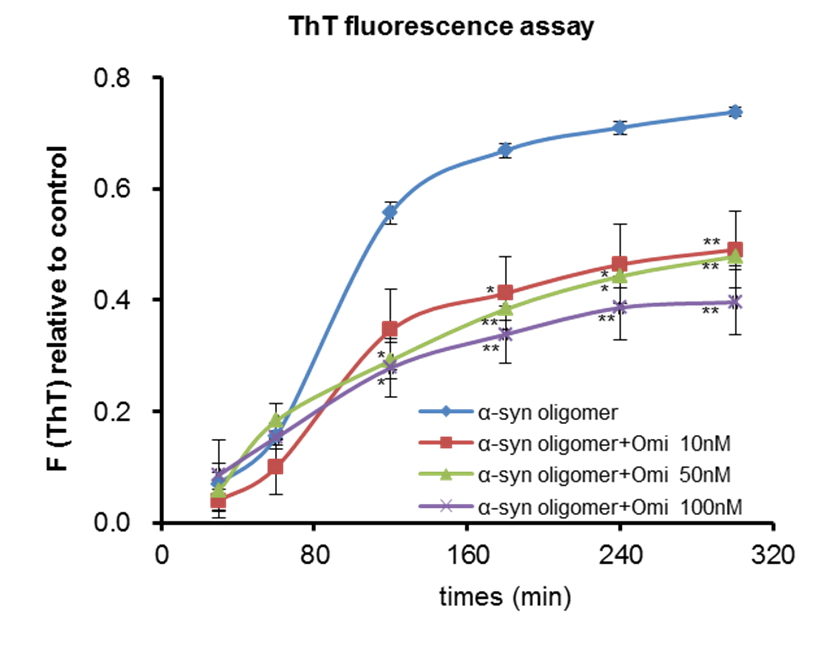
**

**Figure S3.**

**
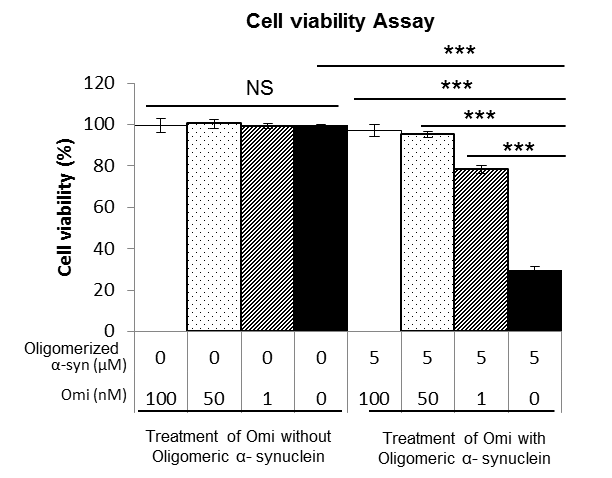
**

**Figure S4.**

**
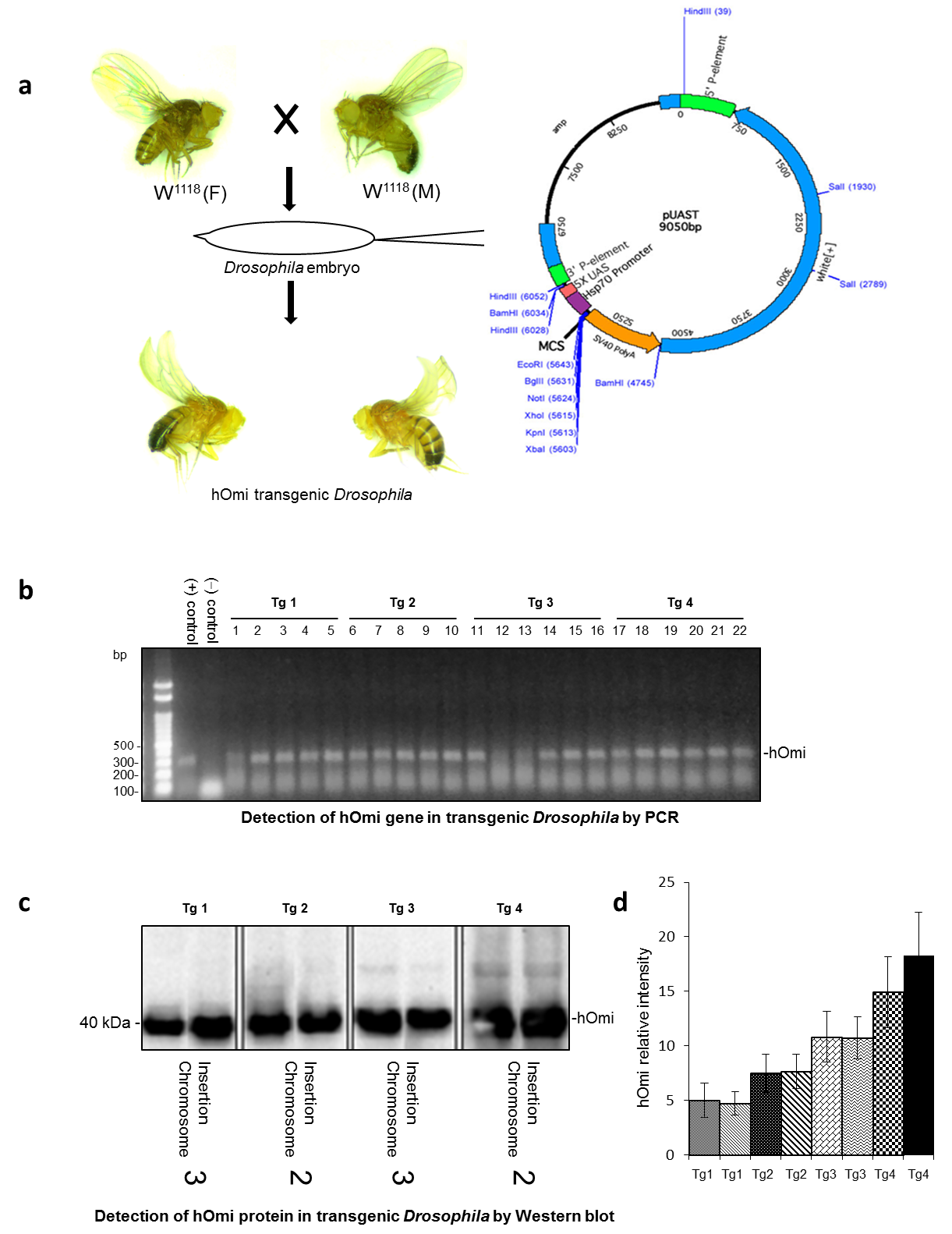
**

**Figure S5.**

**
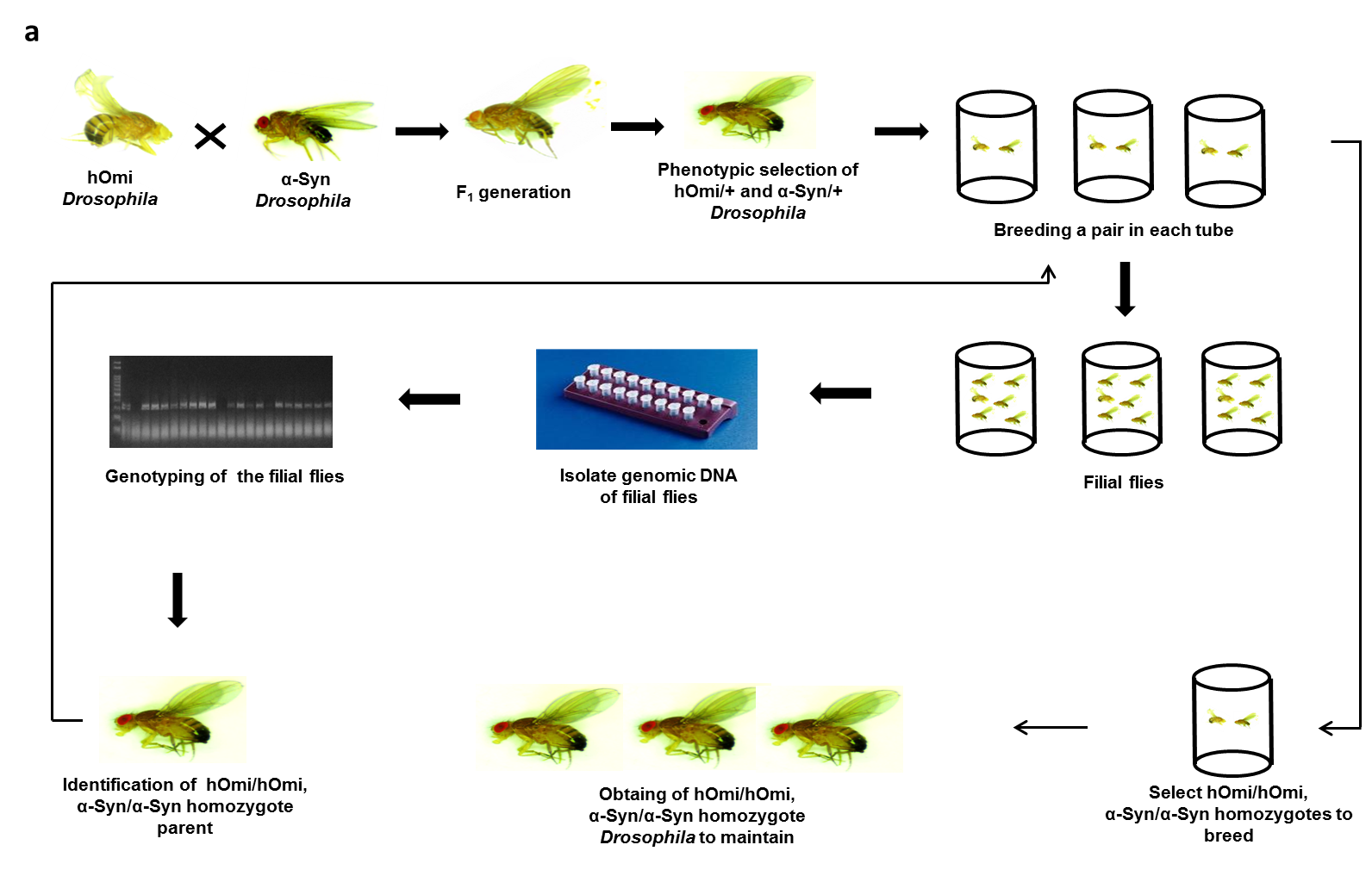
**

**
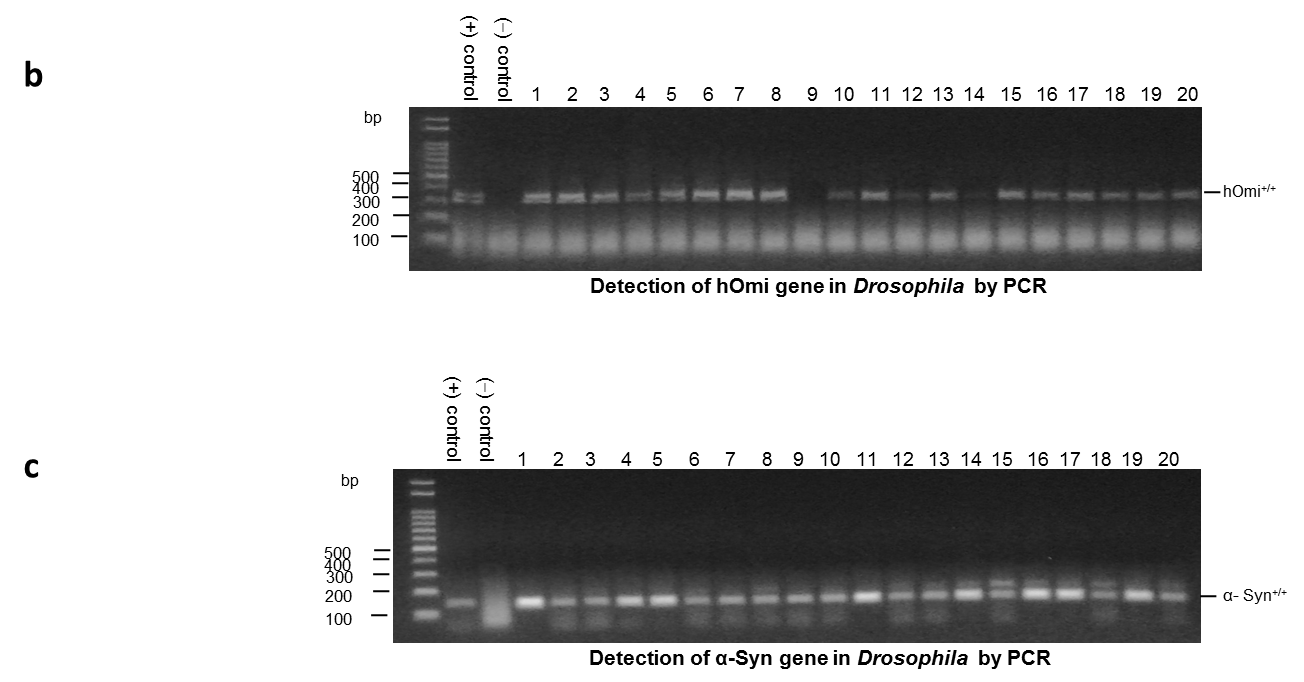
**

**Figure S6.**

**
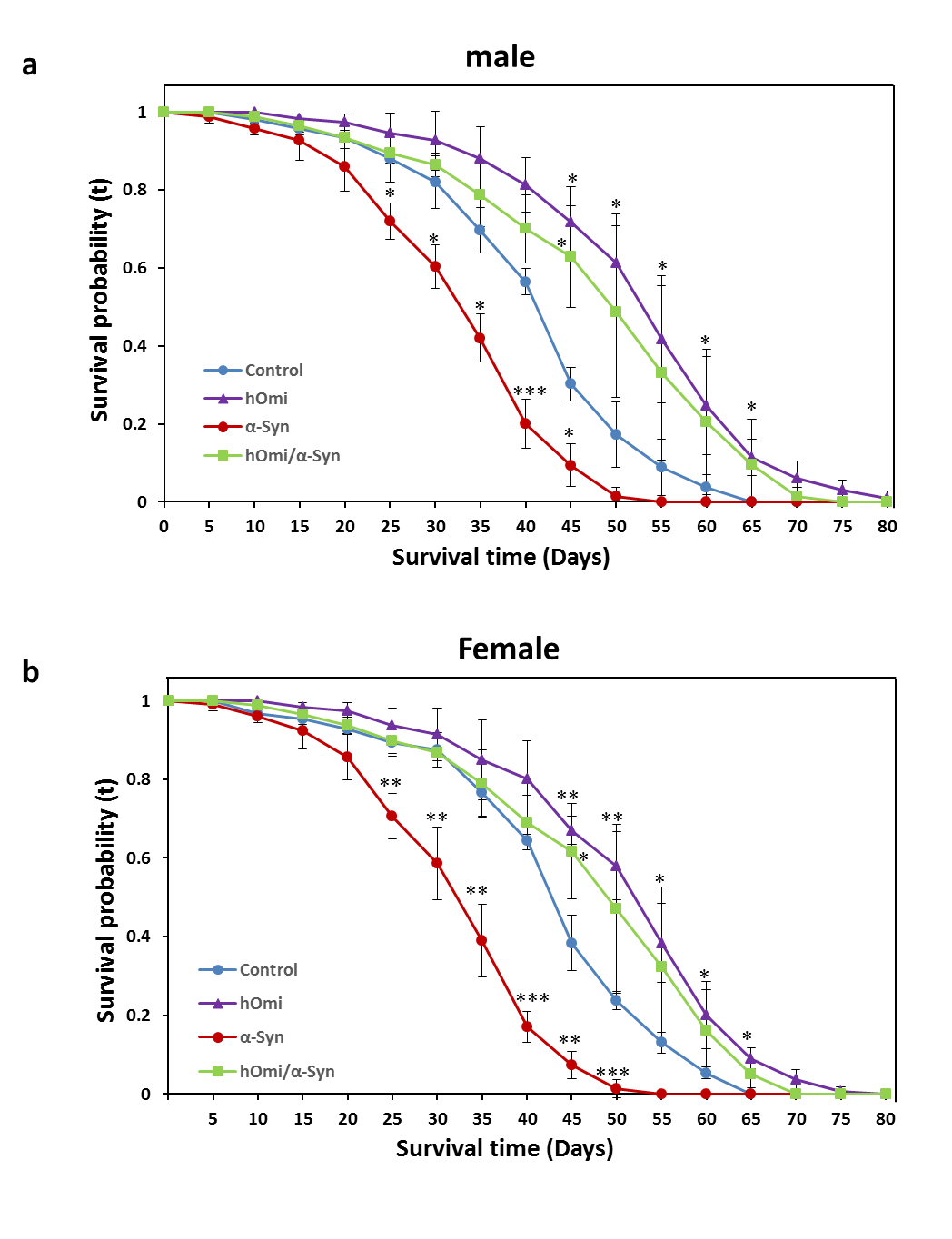
**
